# Proximity to Traffic, Ambient Air Pollution, and Community Noise in Relation to Incident Rheumatoid Arthritis

**DOI:** 10.1289/ehp.1307413

**Published:** 2014-06-06

**Authors:** Anneclaire J. De Roos, Mieke Koehoorn, Lillian Tamburic, Hugh W. Davies, Michael Brauer

**Affiliations:** 1 Department of Environmental and Occupational Health, School of Public Health, Drexel University, Philadelphia, Pennsylvania, USA; 2 School of Population and Public Health, University of British Columbia, Vancouver, British Columbia, Canada

## Abstract

Background: The risk of rheumatoid arthritis (RA) has been associated with living near traffic; however, there is evidence suggesting that air pollution may not be responsible for this association. Noise, another traffic-generated exposure, has not been studied as a risk factor for RA.

Objectives: We investigated proximity to traffic, ambient air pollution, and community noise in relation to RA in the Vancouver and Victoria regions of British Columbia, Canada.

Methods: Cases and controls were identified in a cohort of adults that was assembled using health insurance registration records. Incident RA cases from 1999 through 2002 were identified by diagnostic codes in combination with prescriptions and type of physician (e.g., rheumatologist). Controls were matched to RA cases by age and sex. Environmental exposures were assigned to each member of the study population by their residential postal code(s). We estimated relative risks using conditional logistic regression, with additional adjustment for median income at the postal code.

Results: RA incidence was increased with proximity to traffic, with an odds ratio (OR) of 1.37 (95% CI: 1.11, 1.68) for residence ≤ 50 m from a highway compared with residence > 150 m away. We found no association with traffic-related exposures such as PM_2.5_, nitrogen oxides, or noise. Ground-level ozone, which was highest in suburban areas, was associated with an increased risk of RA (OR = 1.26; 95% CI: 1.18, 1.36 per interquartile range increase).

Conclusions: Our study confirms a previously observed association of RA risk with proximity to traffic and suggests that neither noise levels nor traffic-related air pollutants are responsible for this relationship. Additional investigation of neighborhood and individual correlates of residence near roadways may provide new insight into risk factors for RA.

Citation: De Roos AJ, Koehoorn M, Tamburic L, Davies HW, Brauer M. 2014. Proximity to traffic, ambient air pollution, and community noise in relation to incident rheumatoid arthritis. Environ Health Perspect 122:1075–1080; http://dx.doi.org/10.1289/ehp.1307413

## Introduction

The identification of respirable silica dust (Parks et al. 1999) and smoking (Sugiyama et al. 2010) as causes of rheumatoid arthritis (RA) suggests inhaled pollution as a possible factor in RA pathogenesis. Biologic mechanisms by which these exposures may lead to RA—inflammation, oxidative stress, and immune suppression—have also been correlated with ambient air pollution (Donaldson et al. 2001; Omara et al. 2000), leading to a hypothesis for the role of air pollution in the development of RA.

Traffic, a major source of air pollution, was investigated in relation to RA in the Nurses’ Health Study (NHS) cohort in the United States (Hart et al. 2009). Women who lived ≤ 50 m from a roadway were at 31% increased risk of RA, compared with those who lived ≥ 200 m away. However, there was no risk associated with specific air pollutants, including particulate matter with an aerodynamic diameter ≤ 2.5 μm (PM_2.5_), nitrogen dioxide (NO_2_), and sulfur dixoxide (SO_2_) in the same study population (Hart et al. 2013a). In a case–control study conducted in Sweden, Hart et al. (2013b) found small increases in RA risk associated with SO_2_ exposure in the 10th and 20th years before disease onset and an association with NO_2_ for only autoantibody-negative cases. The same group found no association with PM_10_ (≤ 10 μm in aerodynamic diameter). These initial studies provided some consistency of evidence against PM exposure as a risk factor for RA, but the results were inconsistent regarding other air pollutants.

The association of RA with traffic in the NHS remains unexplained in terms of identification of a causal agent, and Hart et al. (2013a) speculated that road proximity may be a proxy for non–air pollution exposures, such as noise or neighborhood. Noise is a biologically plausible etiologic agent whereby traffic noise may be perceived as stressful and produce an immunologic response (Prasher 2009). An important mechanism for physiologic effects from urban noise is through sleep disturbance (Zaharna and Guilleminault 2010). Stress from major life events has been linked with onset of RA in multiple studies (Herrmann et al. 2000); however, noise as a stressor has not been studied as a cause of RA.

We investigated proximity to traffic, ambient air pollution, and community noise as risk factors for RA in a large population-based study conducted in the Vancouver and Victoria regions of British Columbia (BC), Canada. In addition to providing data on traffic and air pollutants for comparison with previous studies of RA, our investigation of noise generates new information that could help explain the relationship of RA with proximity to roadways.

## Methods

*Study population*. We conducted research for the present study within the Border Air Quality Study cohort (Gan et al. 2011, 2012a; University of British Columbia 2014), in which the study population was identified using birthdate and postal code(s) of residence in linked administrative databases from BC’s universal health insurance system (BC Ministry of Health 2006a, 2006b, 2006c). The BC Ministry of Health and the BC Vital Statistics Agency approved access to the data, and its use was facilitated by Population Data BC (https://www.popdata.bc.ca). All of the study procedures, including access to and linkage of administrative data, were covered under the ethics approval provided by the Behavioural Research Ethics Board of the University of British Columbia (certificate no. H05-80442). The study region encompassed the metropolitan centers of Vancouver and Victoria and surrounding areas in the same airshed. The cohort included 678,361 area residents who were registered with the Canadian health insurance plan, lived in the study area during the 5-year cohort definition period (1994–1998), and were 45–84 years of age at the start of the cohort follow-up period (1 January 1999).

We used a nested case–control design for this study of RA in order to evaluate risk in relation to a consistent window of exposure for each member of the study population (5 years before the diagnosis date). Cases and controls were selected from cohort members considered to be “at-risk” for developing RA at the start of the 4-year follow-up period (1 January 1999 through 31 December 2002) and included 640,041 persons with no diagnosis code for RA [*International Classification of Diseases, Version 9* (ICD-9); code 714 [rheumatoid arthritis and other inflammatory polyarthropathies (codes 714.0–714.9); National Center for Health Statistics 1998] recorded in fee-for-service physician claims or for hospitalizations during the cohort definition period.

Cases. We identified RA cases by the ICD-9 code (714.0–714.9) listed for an outpatient or inpatient visit. The diagnosis date of each case (the index date) was defined by the first appearance of the relevant ICD-9 code. We used three definitions of RA:

RA-ICD-9: three or more ICD-9 codes for RA recorded during follow-up (with two codes > 30 days apart)
RA-prescription: two or more ICD-9 codes for RA recorded during follow-up (> 30 days apart) and one or more prescriptions (BC Ministry of Health 2012a) for a disease-modifying antirheumatic drug (DMARD) or steroids (oral or injectable, not topical) after the first ICD-9 code
RA-specialist: two or more ICD-9 codes for RA recorded during follow-up (> 30 days apart) and one or more of these was for a visit to a physician specialist (i.e., a rheumatologist or internist).

Identification of RA patients based on two or more ICD-9 codes at least 30 days apart had 92% positive predictive value (PPV) compared with patient self-report within a large U.S. West Coast insurance company (MacLean et al. 2001). We required three or more ICD-9 codes to increase the specificity of our RA-ICD-9 definition. Our RA-prescription definition was based on validation studies in other populations that have shown information on medications in combination with ICD-9 codes (Singh et al. 2004) or self-report (Walitt et al. 2008) to further improve the specificity of case classification. We allowed either steroid or DMARD medications for the RA-prescription definition because DMARD use among RA cases in Canada was considered inappropriately low in a previous study of administrative billing data (Lacaille et al. 2005). Our RA-specialist case definition was based on the BC Ministry of Health Guidelines recommendation for early referral to a specialist when a new diagnosis of RA is suspected (BC Ministry of Health 2012b).

Controls. Ten controls per case were selected from the cohort remaining at risk on the index date of each RA case, matched to cases by age and sex.

*Exposure assessment*. Exposure estimates were individually assigned to each member of the study population for a 5-year period before their index date, based on the six-digit postal code of their residence(s). The size of an area covered by a residential postal code depends on the population density of the area; on average, a postal code includes 35 persons. A total of 97.5% of postal codes in the at-risk cohort’s residential histories were georeferenced to the block-face (one side of a city block) level.

Traffic. We used the commercially available DMTI CanMap road network to identify road locations and attributes (DMTI Spatial, Markham, Ontario, Canada). We defined “highways” as combined DMTI road categories 1 (expressways, usually four lanes) and 2 (principal highways, multilane conduits for intracity traffic), with average traffic counts for the two road types of 114,000 and 21,000 vehicles/day, respectively (Setton et al. 2005). “Major roads” were defined as DMTI road categories 3 (secondary highways, typically thoroughfares with multiple lanes and large traffic capacity) and 4 (major roads, used for shorter trips within the city), with average traffic counts of 15,000 and 18,000 vehicles/day. For each road type, we coded residential proximity in categories of ≤ 50 m or 50–150 m from the nearest road. Members of the study population who lived in multiple residences during the exposure period were categorized according to the proximity at which they lived the longest. Proximity variables were compared with an unexposed category that included those who lived > 150 m from the road type during the exposure period.

Air pollution. For each member of the study population, monthly air pollutant levels were averaged over their residences(s) during the exposure period using two different approaches.

We used land-use regression (LUR) models to estimate individual residential exposures to black carbon, PM_2.5_, NO_2_, and nitrogen oxide (NO) in 2003 (Brauer et al. 2008; Henderson et al. 2007; Larson et al. 2009). The models were based on NO and NO_2_ measurements at 116 sites, PM_2.5_ measurements at a subset of 25 locations, and mobile monitoring of black carbon concentrations at a subset of 39 sites. We combined these measurements with geographic variables to develop high spatial resolution (10-m) LUR models. The coefficient of determination (*R*^2^) of the models ranged from 0.52 for PM_2.5_ to 0.62 for NO. The models were used to generate a smooth spatial surface of predicted monthly concentrations for each air pollutant at each postal code during the study period. Previous work within our study region showed that the spatial pattern of pollution was adequately stable for extrapolation of an LUR model over a 7-year period (Wang et al. 2012).

In a second air pollution assessment approach, pollutant concentrations measured at regulatory monitors [*n* = 24 monitors for PM_10_, 26 for nitrogen oxides, 27 for ozone (O_3_), 20 for SO_2_, 21 for carbon monoxide (CO)] were assigned to postal codes by inverse-distance weighting (IDW) of the three closest monitors ≤ 50 km of the postal code for each day (Marshall et al. 2008). A day was set to missing if there were > 6 hr missing from the monitoring data for that day. From the daily averages, monthly average exposures were computed for each postal code area. A month was considered missing if there was a gap of > 5 consecutive days or if there were > 10 missing days for that month.

Noise. We used noise prediction software (Computer Aided Noise Abatement; Datakustik, Greifenberg, Germany) to estimate annual average community noise levels at each residence during the study period (Gan et al. 2012b). Because of model inputs required, noise exposure was only estimated for residences in metropolitan Vancouver. Noise exposure was based on data from 2003, including road type (Setton et al. 2005), traffic volume from a transportation planning model (EMME/2; INRO Consultants, Montreal, Quebec, Canada), railway data (e.g., type of train, velocity, and frequency), aircraft data from noise exposure forecasts produced by Vancouver International Airport Authority (Transport Canada 2005), and building heights and footprints. The annual A-weighted equivalent continuous noise level was calculated for a 10 × 10 m grid that was then averaged for each postal code area. This metric integrated noise levels during the day, evening, and night, with 5-dB(A) weighting added to evening noise and 10-dB(A) weighting added to night noise to reflect increased sensitivity of residents to noise during these periods. For each member of the study population, annual noise levels were averaged over their residence(s) during the exposure period.

*Statistical analyses*. Data analyses were conducted using SAS version 9.3 (SAS Institute Inc., Cary, NC, USA). We generated odds ratios (ORs) as estimates of relative risks (RRs) from conditional logistic regression models (Breslow and Day 1980), accounting for the age- and sex-matched design. We adjusted for potential confounding by neighborhood-level socioeconomic status (SES) using the median income level of the residential postal code from the 2001 Statistics Canada Census (Statistics Canada 2001), split by quintiles. There was no confounding by neighborhood SES in overall analyses; however, we retained this variable in all models based on evidence of weak confounding in subgroup analyses. Each road type was modeled separately, including the two proximity categories in one model with the unexposed category as the referent. We also repeated the traffic analyses when excluding persons who moved during the 5-year exposure period. ORs for associations with continuous air pollutant and noise variables were estimated for an increase across the interquartile range (IQR). In addition, we estimated ORs for quintiles of exposure, based on control exposure distributions. To evaluate the consistency of our findings, we conducted analyses within subgroups of age (< 65 and ≥ 65 years), sex, and neighborhood SES (lowest two vs. highest three quintiles).

We did not have individual-level data on smoking; however, information existed for 2,030 persons in our at-risk cohort who had consented to link their 2000–2001 Canadian Community Health Survey responses (Statistics Canada 2002) to their medical services records for research purposes. We tabulated the prevalence of smoking by air pollution and roadway exposures in our at-risk cohort. From the smoking frequencies, in combination with the published association between smoking and RA (Sugiyama et al. 2010), we used the Episens program as described by Orsini et al. (2008) to estimate the direction and magnitude of confounding by smoking.

## Results

The distribution of air pollution and noise exposures and the correlation between these variables are shown in Table 1. NO and NO_2_ were strongly correlated (*r* = 0.82), and NO_2_ was also moderately correlated with black carbon, PM_2.5_, and SO_2_. O_3_ was negatively correlated with all other air pollutants and noise, with the strongest negative correlations for SO_2_, CO, and NO_2_. Noise was not highly correlated with any of the air pollutants; the strongest correlations were with NO (*r* = 0.40) and NO_2_ (*r* = 0.33). Members of the study population who lived ≤ 50 m from a highway had higher exposures than those who lived > 150 m away, for noise [mean, 71.3 vs. 63.0 dB(A)], NO (44.9 vs. 29.8 μg/m^3^), and black carbon (1.60 vs. 1.24 μg/m^3^). There was little difference in the other pollutants by proximity to traffic (see Supplemental Material, Table S1).

**Table 1:** Air pollution and noise exposure distributions and correlations in the at-risk cohort during the cohort definition period (1994–1998).

Exposure	Mean ± SD	Median (IQR)	Range	LUR				IDW				Noise
				NO	NO_2_	Black carbon	PM_2.5_	PM_10_	O_3 _	CO	SO_2_	
NO-LUR (μg/m^3^)	30.8 ± 11.6	27.8 (23.8–34.5)	11.4–126	1	0.82	0.55	0.41	0.06	–0.31	0.38	0.36	0.40
NO_2_-LUR (μg/m^3^)	29.1 ± 5.2	28.4 (25.6–31.9)	15.1–57.5		1	0.63	0.61	0.03	–0.46	0.49	0.52	0.33
Black carbon-LUR (μg/m^3^)	1.3 ± 0.6	1.1 (0.9–1.5)	0–4.3			1	0.51	–0.06	–0.40	0.41	0.49	0.23
PM_2.5_-LUR (μg/m^3^)	4.7 ± 2.4	4.3 (3.2–5.9)	0–10.2				1	–0.01	–0.28	0.35	0.35	0.12
PM_10_-IDW (μg/m^3^)	13.9 ± 0.6	13.8 (13.5–14.3)	10.9–16.0					1	–0.22	0.28	–0.10	0.05
O_3_-IDW (μg/m^3^)	27.1 ± 5.0	26.5 (23.7–31.2)	13.7–38.3						1	–0.92	–0.84	–0.14
CO-IDW (μg/m^3^)	741 ± 154	719 (640–841)	397–1,169							1	0.74	0.11
SO_2_-IDW (μg/m^3^)	6.5 ± 2.8	5.6 (4.5–8.4)	0.4–15.9								1	0.11
Noise [dB(A)]	63.5 ± 5.1	62.6 (59.9–66.8)	< 25–98.5									1
Data are correlation coefficients unless otherwise indicated.												

We identified 3,333 RA-ICD-9, 2,692 RA-prescription, and 1,911 RA-specialist cases among the 640,041 at-risk cohort members during 4 years of follow-up (Table 2). These numbers are consistent with the expected number of roughly 2,100 incident cases, estimated from published age-stratified incidence rates (Doran et al. 2002). There was a large overlap of individuals among the three case definitions, with 82% and 83% of RA-specialist cases also counted as RA-prescription or RA-ICD-9 cases, respectively, and 81% of RA-prescription cases also counted as RA-ICD-9 cases. Compared to the at-risk cohort, overall, cases were older at the study baseline and more likely to be female. The distribution of neighborhood SES was fairly comparable between cases and the at-risk cohort.

**Table 2:** Characteristics of the study population at baseline (1999) [*n* (%)].

Characteristic	At-risk cohort (*n* = 640,041)	RA-ICD-9 (*n* = 3,333)	RA-prescription (*n* = 2,692)	RA-specialist (*n* = 1,911)
Age (years)
45–54	257,061 (40.2)	934 (28.0)	687 (25.5)	567 (29.7)
55–64	160,246 (25.0)	820 (24.6)	663 (24.6)	503 (26.3)
65–74	137,130 (21.4)	955 (28.7)	804 (29.9)	534 (27.9)
75–84	85,604 (13.4)	624 (18.7)	538 (20.0)	307 (16.1)
Sex
Female	335,062 (52.4)	2,218 (66.6)	1,854 (68.9)	1,307 (68.4)
Male	304,979 (47.6)	1,115 (33.4)	838 (31.3)	604 (31.6)
Neighborhood SES (quintile)
1	126,005 (19.7)	732 (22.0)	580 (21.6)	378 (19.8)
2	125,335 (19.6)	748 (22.4)	551 (20.5)	380 (19.9)
3	123,928 (19.4)	626 (18.8)	510 (19.0)	354 (18.5)
4	131,334 (20.5)	624 (18.7)	539 (20.0)	404 (21.1)
5	131,871 (20.6)	596 (17.9)	506 (18.8)	391 (20.5)
Missing	1,568 (0.2)	7 (0.2)	6 (0.2)	4 (0.2)

**Figure 1:**
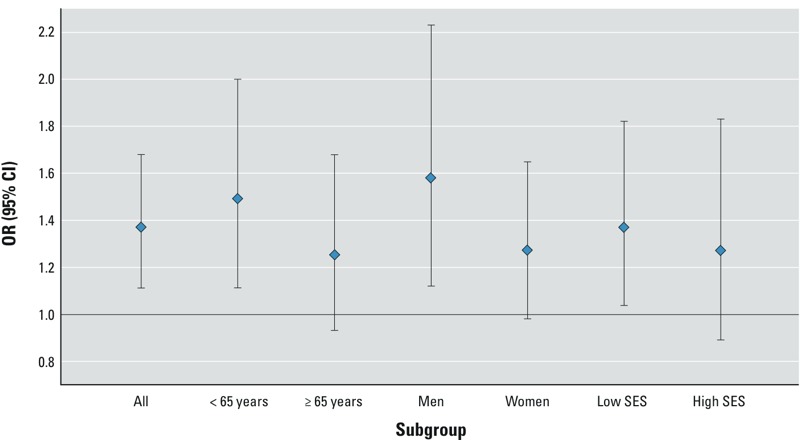
Risk of incident RA (RA-prescription definition) in relation to residence ≤ 50 m from a highway (vs. > 150 m from a highway) during 5 years before diagnosis.

Residential proximity to a highway was associated with RA for each of the three case definitions (Table 3), with estimated risk increases of 37–39% for residence ≤ 50 m and 7–24% for 50–150 m, during the 5 years before diagnosis. ORs were somewhat attenuated when we excluded those who moved during the exposure period; for example, instead of a 37% increased risk, there was a 20% increased risk (95% CI: –13%, 65%) of RA-prescription diagnosis associated with continuous residence ≤ 50 m from a highway (70% of cases and 78% of controls did not move). There was no association with proximity to a major road. Risk increases with proximity to highways were observed across all subgroups of sex, age, and neighborhood SES, although not all elevations were statistically significant. For example, residence ≤ 50 m from a highway was associated with incidence of RA-prescription diagnosis in all subgroups (Figure 1), with ORs ranging from 1.19 to 1.61 and statistically significant elevations estimated for the younger-age, male, and lower-SES subgroups. Similar profiles were obtained for the other case definitions except for the RA-specialist case definition, for which the association between residence ≤ 50 m from a highway and risk of RA was seen in lower-SES neighborhoods (OR = 1.73; 95% CI: 1.23, 2.44) but not with higher SES (OR = 1.04).

**Table 3:** Risk of incident RA in relation to residential proximity to traffic during 5 years before diagnosis [ORs (95% CIs)].

Exposure	RA-ICD-9			RA-prescription			RA-specialist		
	Percent cases (*n* = 3,226)	Percent controls (*n* = 33,256)	OR (95% CI)	Percent cases (*n* = 2,686)	Percent controls (*n* = 26,857)	OR (95% CI)	Percent cases (*n* = 1,907)	Percent controls (*n* = 19,066)	OR (95% CI)
Distance from highway
> 150 m	86.9	89.3	1.0 (referent)	87.0	89.4	1.0 (referent)	87.8	89.0	1.0 (referent)
> 50–150 m	9.1	7.8	1.17 (1.03, 1.33)	9.1	7.6	1.24 (1.08, 1.43)	8.3	8.1	1.07 (0.90, 1.27)
≤ 50 m	3.9	2.8	1.39 (1.16, 1.68)	4.0	3.0	1.37 (1.11, 1.68)	3.9	2.9	1.37 (1.07, 1.76)
Distance from major road
> 150 m	59.7	61.2	1.0 (referent)	60.6	61.5	1.0 (referent)	61.9	61.7	1.0 (referent)
> 50–150 m	26.8	25.9	1.05 (0.97, 1.14)	25.7	25.5	1.02 (0.93, 1.12)	26.0	25.4	0.94 (0.81, 1.09)
≤ 50 m	13.5	12.8	1.07 (0.96, 1.19)	13.7	13.0	1.07 (0.95, 1.20)	12.1	12.9	1.02 (0.92, 1.14)
Members of the study population who lived ≤ 150 m from a highway/major road during the 5-year exposure period were categorized according to proximity at which they lived the longest. ORs were adjusted for age, sex, and neighborhood SES.									

**Figure 2:**
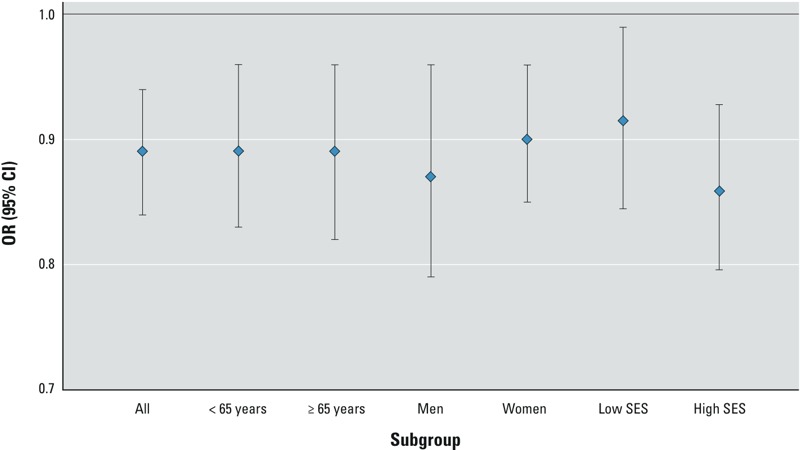
Risk of incident RA (RA-prescription definition) in relation to NO_2_ (per IQR increase) during 5 years before diagnosis.

ORs for the association between ambient air pollutants and RA are shown in Table 4 (results for NO and NO_2_ assessed by IDW did not differ importantly from the LUR results; see Supplemental Material, Table S2). The risk of developing RA was not increased with exposure to NO, NO_2_, black carbon, PM_2.5_, PM_10_, CO, or SO_2_. Conversely, many of the ORs were < 1. Analyses of exposure quintiles for these pollutants generally showed monotonic trends of decreasing risk by increasing category of exposure (see Supplemental Material, Table S3). Inverse associations were quite consistent in subgroup analyses by age, sex, and neighborhood SES (e.g., Figure 2 shows RA-prescription in relation to NO_2_).

**Table 4:** Risk of incident RA in relation to ambient air pollution and community noise during 5 years before diagnosis [ORs (95% CIs)] per IQR increase.

Exposure	RA-ICD-9			RA-prescription			RA-specialist		
	Cases (*n*)	Controls (*n*)	OR (95% CI) [IQR]	Cases (*n*)	Controls (*n*)	OR (95% CI) [IQR]	Cases (*n*)	Controls (*n*)	OR (95% CI) [IQR]
NO-LUR (μg/m^3^)	3,280	33,234	0.99 (0.95, 1.02) [10.6]	2,659	26,846	0.96 (0.92, 1.00) [10.8]	1,883	19,059	0.96 (0.91, 1.00) [10.6]
NO_2_-LUR (μg/m^3^)	3,278	33,229	0.95 (0.90, 0.99) [6.3]	2,657	26,842	0.89 (0.84, 0.94) [6.3]	1,881	19,059	0.90 (0.85, 0.96) [6.3]
Black carbon-LUR (μg/m^3^)	3,138	32,159	0.97 (0.93, 1.01) [0.62]	2,553	25,935	0.94 (0.90, 0.98) [0.61]	1,818	18,420	0.92 (0.87, 0.97) [0.62]
PM_2.5_-LUR (μg/m^3^)	3,175	32,304	0.96 (0.91, 1.00) [2.7]	2,567	26,144	0.93 (0.88, 0.98) [2.7]	1,819	18,518	0.92 (0.87, 0.98) [2.7]
PM_10_-IDW (μg/m^3^)	2,712	27,208	0.91 (0.88, 0.95) [0.87]	2,135	21,850	0.90 (0.86, 0.94) [0.87]	1,653	15,709	0.91 (0.86, 0.96) [0.87]
O_3_-IDW (μg/m^3^)	3,055	30,698	1.15 (1.08, 1.23) [8.4]	2,454	24,791	1.26 (1.18, 1.36) [8.6]	1,724	17,636	1.07 (0.98, 1.16) [8.4]
CO-IDW (μg/m^3^)	2,826	28,269	0.87 (0.82, 0.91) [169]	2,249	22,807	0.83 (0.78, 0.88) [169]	1,633	16,274	0.86 (0.80, 0.92) [169]
SO_2_-IDW (μg/m^3^)	3,082	30,963	0.90 (0.86, 0.94) [3.1]	2,477	25,011	0.84 (0.79, 0.89) [3.1]	1,733	17,761	0.88 (0.82, 0.93) [3.1]
Noise [dB(A)]	2,188	22,734	1.03 (0.97, 1.09) [6.9]	1,711	18,346	1.00 (0.93, 1.07) [7.0]	1,315	13,173	0.96 (0.88, 1.04) [6.9]
ORs were adjusted for age, sex, and neighborhood SES.									

**Figure 3:**
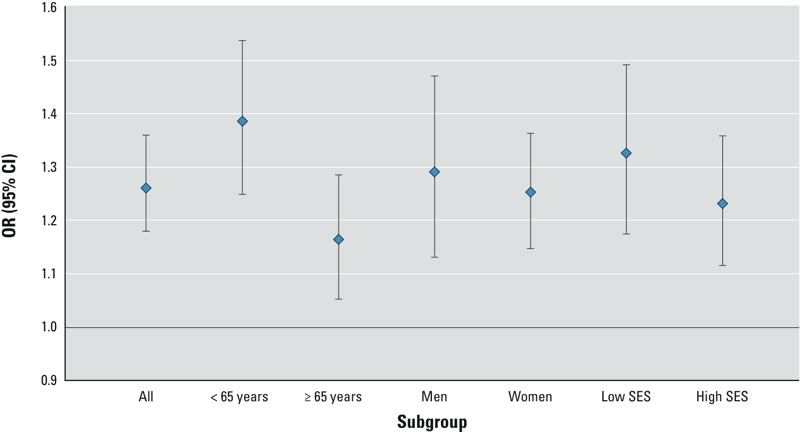
Risk of incident RA (RA-prescription definition) in relation to O_3_ (per IQR increase) during 5 years before diagnosis.

Ground-level O_3_ was associated with 15% and 26% increased risk of RA (for RA-ICD-9 and RA-prescription, respectively) for an increase across the IQR (Table 4), and 29% and 56% increased risk with O_3_ level in the highest versus lowest quintile category (see Supplemental Material, Table S3). Cases identified using the RA-specialist definition showed significant associations with O_3_ that increased by quintile of exposure, but diminished in the highest category. Risk increases were fairly consistent across age, sex, and neighborhood SES subgroups (Figure 3 shows RA-prescription results), although the association was diminished for ages ≥ 65 years.

We found no association of community noise exposure with risk of RA, overall (Table 4), or in subgroup analyses.

Among the small subgroup with prior health survey data, smoking distribution differed by the exposures we studied. Classification as an “ever smoker” was higher among those who lived ≤ 50 m from a highway (69% vs. 60% in those living farther away). Smoking was less frequent among members of the study population in areas with the highest levels of NO_2_ and other pollutants for which we observed inverse associations, and smoking was more frequent among those in areas with the highest O_3_ levels. Based on the smoking distributions and the association reported in a recent meta-analysis for ever-smoked (RR = 1.40) in relation to RA (Sugiyama et al. 2010), sensitivity analyses suggested that smoking is unlikely to be an important confounder in our study. Bias adjustment for confounding by ever-smoking resulted in only a 2–3% change in the risk estimates for proximity to traffic (e.g., a change in the OR for ≤ 50 m from highway from 1.37 to 1.33), and 0 to 4% change in ORs for the highest versus lowest quintiles of NO_2_ or O_3_. Confounding from smoking may be stronger among lower-SES members of the study population, among which the prevalence of smoking was higher and differed by proximity to highways (77% in those ≤ 50 m from highway vs. 62% in those farther away). Nevertheless, there was at most a 5% change in the risk estimates for proximity to traffic in the lower SES subgroup with bias adjustment for smoking.

## Discussion

Through the use of the established population–based Border Air Quality Study cohort with state-of-the-art exposure modeling, we were able to efficiently evaluate the effects of proximity to traffic, ambient air pollution, and community noise on the risk of developing RA. Use of health records linked to postal codes in the Vancouver and Victoria, BC, metropolitan areas allowed us to identify the sizable number of cases needed for investigation of possibly subtle effects of environmental exposures on RA.

We observed an increased risk of RA with residential proximity to traffic and the largest risk increases were seen in association with road types of higher traffic volume and with greater proximity, suggesting a dose–response pattern. Our findings are similar to those from the NHS, where RA was associated with proximity to roadways (Hart et al. 2009) but not with specific traffic-related air pollutants such as NO_2_ or PM_2.5_ (Hart et al. 2013a). Another exposure from traffic—noise—was tested for the first time, and was not associated with RA in our study. The increased risk we observed with traffic could be due to confounding by risk factors for RA that may be more prevalent close to roadways, such as low SES, nonwhite race, and smoking. The health service records do not contain information on these variables; however, there was little confounding in our study due to neighborhood-level income, and sensitivity analyses suggested that smoking was unlikely to be an important confounder. Indeed, adjustment for multiple individual-level covariates in the NHS indicated little confounding of the association with proximity to roadways. Nevertheless, inconsistencies in our findings (diminished association among non-movers and association limited to lower SES neighborhoods for one case definition) suggest that other, nontraffic exposures underlie the association.

The present study and the previous two studies by Hart et al. (2013a, 2013b) did not observe any risk increase with PM_2.5_ or PM_10_, providing evidence against PM air pollution as a risk factor for RA. We likewise did not observe increased risk with NO_2_ or SO_2_, contrary to the findings of the Swedish study (Hart et al. 2013b). In fact, we observed inverse associations with certain air pollutants including NO_2_, SO_2_, and PM_10_ (i.e., suggested protective effects) that were counter to our hypothesis. The range and variability of many air pollutants in the Vancouver region is lower than in other major cities, which may have hindered detection of risks limited to higher exposure levels. For example, the SO_2_ IQR was larger in both the Swedish study (8 μg/m^3^) and the NHS (14 μg/m^3^) (Hart et al. 2013a) than in the present study (3.1 μg/m^3^). Furthermore, it is possible that the induction period for these exposures is longer than the 5 years evaluated here, as associations with NO_2_ and SO_2_ in the Swedish study were strongest for exposure in the 10th year before disease onset. Discrepant results between the three studies for SO_2_ and NO_2_ leave an open question about the role of these pollutants in RA.

Noise has not been studied in relation to RA in humans. In rats, 90- to 95-dB noise exposure for a 1-hr period for 7 days was associated with earlier onset and increased severity of collagen-induced arthritis (Rogers et al. 1983), but there are few other existing data for evaluating the relationship. The noise metric we used represented an average community noise level across day, evening, and night hours, and ranged from < 25 to 98.5 dB(A). This metric was associated with coronary heart disease mortality in a previous investigation within the cohort (Gan et al. 2012a), with 9% increased risk per 10-dB(A) noise level that was independent of traffic-related air pollutants. We did not see any association with the risk of RA. Aside from the possibility of a true null association, it is also possible that average noise level does not capture the aspects of noise that could be etiologically relevant for RA, such as peak levels.

Ground-level O_3_ was associated with increased risk of RA in the present study. The previous epidemiologic studies of RA have not reported on O_3_ (Hart et al. 2013a, 2013b). O_3_ causes its toxic effects through oxidation or peroxidation of biomolecules—directly or via free-radical reactions—potentially causing altered methylation or protein binding sites in DNA and thus generating autoantigens that may lead to autoimmunity (Kannan 2006; Kirkham et al. 2011). O_3_ is formed in the ambient environment through the reaction of hydrocarbons with nitrogen oxides in the presence of ultraviolet energy. Because O_3_ is itself reactive, areas with high levels of nitrogen oxides and locations close to traffic tend to have low O_3_ levels. The areas with the highest O_3_ levels in our study region were suburban neighborhoods with higher SES (Marshall et al. 2009). Confounding from factors spatially associated with these neighborhoods may be of concern, such as white race/ethnicity, obesity, and physical inactivity (Marshall et al. 2009). Nevertheless, due to limited knowledge about risk factors for RA, few variables are readily identifiable as likely confounders.

Because the Border Air Quality Study was enumerated using health insurance registration records, it is a comprehensive, population-based sample that is less subject to selection biases than a study requiring active participation. Furthermore, the near-complete health coverage in Canada allowed us to identify RA cases in a population not influenced by access to health care. These strengths are unfortunately paired with weaknesses. Identification of incident RA cases from information in health service billing records is inherently imperfect, and although our case definitions were informed by validation in other populations, case diagnoses in our study were not confirmed. Our data were further limited by a lack of certain details that would be valuable for distinguishing case groups, such as autoantibody-positivity [for rheumatoid factor (RF) or anticitrullinated peptide antibodies (ACPA)]. Such details may be important because the association with proximity to roadways in the NHS was stronger for RF-positive cases (Hart et al. 2009) and the Swedish case–control study found risk increases associated with NO_2_ for ACPA-negative cases only (Hart et al. 2013b).

The present study capitalized on extensive exposure modeling conducted by a multidisciplinary team, in which air pollution levels were assigned to individual members of the study population using methods that incorporate both intraurban spatial contrasts and temporal variability. Nevertheless, there are limitations to our models. Exposures were estimated at residential locations only, and therefore did not include air pollutant exposure inputs from workplace locations and time spent in traffic. Despite this potential misclassification, the majority of a person’s time is spent at their residence (Leech et al. 2002), making ambient exposure in residential neighborhoods an important regulatory target. Another concern is that there are indoor as well as outdoor sources of the pollutants we studied. Correlations between ambient and personal exposures have been shown to be stronger for PM than for the gaseous pollutants, including O_3_ (Sarnat et al. 2001). We understand this potential measurement error, and stress that our finding of an O_3_ association with RA should be investigated in other study populations.

## Conclusions

Our findings confirm a previously observed association of RA risk with proximity to traffic, and suggest that neither noise levels nor traffic-related air pollutants are responsible for this relationship. Additional investigation of neighborhood and individual correlates of residence near roadways may provide new insight into risk factors for RA.

## Supplemental Material

(264 KB) PDF

## References

[r1] BC Ministry of Health. (2006a). Consolidation File (BC Ministry of Health Services Registration & Premium Billing (R&PB) files).. https://www.popdata.bc.ca/data/internal/demographic/consolidationfile.

[r2] BC Ministry of Health. (2006b). Discharge Abstracts Database (Hospital Separations).. https://www.popdata.bc.ca/data/internal/health/dad.

[r3] BC Ministry of Health. (2006c). Medical Services Plan (MSP) Payment Information File.. https://www.popdata.bc.ca/data/internal/health/msp.

[r4] BC Ministry of Health. (2012a). PharmaNet.. http://www.health.gov.bc.ca/pharmacare/pharmanet/netindex.html.

[r5] BC Ministry of Health. (2012b). Rheumatoid Arthritis: Diagnosis, Management and Monitoring.. http://www.bcguidelines.ca/guideline_ra.html.

[r6] Brauer M Lencar C Tamburic L Koehoorn M Demers P Karr C 2008 A cohort study of traffic-related air pollution impacts on birth outcomes. Environ Health Perspect 116 680 686; 10.1289/ehp.10952 18470315 PMC2367679

[r7] Breslow NE Day NE 1980 Statistical Methods in Cancer Research, Vol 1. The Analysis of Case–Control Studies. IARC Sci Publ (32 5 338 7216345

[r8] Donaldson K, Stone V, Seaton A, MacNee W (2001). Ambient particle inhalation and the cardiovascular system: potential mechanisms.. Environ Health Perspect.

[r9] Doran MF, Pond GR, Crowson CS, O’Fallon WM, Gabriel SE (2002). Trends in incidence and mortality in rheumatoid arthritis in Rochester, Minnesota, over a forty-year period.. Arthritis Rheum.

[r10] Gan WQ, Davies HW, Koehoorn M, Brauer M (2012a). Association of long-term exposure to community noise and traffic-related air pollution with coronary heart disease mortality.. Am J Epidemiol.

[r11] Gan WQ Koehoorn M Davies HW Demers PA Tamburic L Brauer M 2011 Long-term exposure to traffic-related air pollution and the risk of coronary heart disease hospitalization and mortality. Environ Health Perspect 119 501 507; 10.1289/ehp.1002511 21081301 PMC3080932

[r12] Gan WQ, McLean K, Brauer M, Chiarello SA, Davies HW (2012b). Modeling population exposure to community noise and air pollution in a large metropolitan area.. Environ Res.

[r13] Hart JE, Kallberg H, Laden F, Bellander T, Costenbader KH, Holmqvist M (2013b). Ambient air pollution exposures and risk of rheumatoid arthritis: results from the Swedish EIRA case–control study.. Ann Rheum Dis.

[r14] Hart JE, Kallberg H, Laden F, Costenbader KH, Yanosky JD, Klareskog L (2013a). Ambient air pollution exposures and risk of rheumatoid arthritis.. Arthritis Care Res (Hoboken).

[r15] Hart JE Laden F Puett RC Costenbader KH Karlson EW 2009 Exposure to traffic pollution and increased risk of rheumatoid arthritis. Environ Health Perspect 117 1065 1069; 10.1289/ehp.0800503 19654914 PMC2717131

[r16] Henderson SB, Beckerman B, Jerrett M, Brauer M (2007). Application of land use regression to estimate long-term concentrations of traffic-related nitrogen oxides and fine particulate matter.. Environ Sci Technol.

[r17] Herrmann M, Schölmerich J, Straub RH (2000). Stress and rheumatic diseases.. Rheum Dis Clin North Am.

[r18] Kannan S 2006 Free radical theory of autoimmunity. Theor Biol Med Model 3 22; 10.1186/1742-4682-3-22 16759382 PMC1508139

[r19] Kirkham PA, Caramori G, Casolari P, Papi AA, Edwards M, Shamji B (2011). Oxidative stress-induced antibodies to carbonyl-modified protein correlate with severity of chronic obstructive pulmonary disease.. Am J Respir Crit Care Med.

[r20] Lacaille D, Anis AH, Guh DP, Esdaile JM (2005). Gaps in care for rheumatoid arthritis: a population study.. Arthritis Rheum.

[r21] Larson T, Henderson SB, Brauer M (2009). Mobile monitoring of particle light absorption coefficient in an urban area as a basis for land use regression.. Environ Sci Technol.

[r22] Leech JA, Nelson WC, Burnett RT, Aaron S, Raizenne ME (2002). It’s about time: a comparison of Canadian and American time-activity patterns.. J Expo Anal Environ Epidemiol.

[r23] MacLean CH Park GS Traina SB Liu HH Hahn BH Paulus HE 2001 Positive predictive value (PPV) of an administrative data-based algorithm for the identification of patients with rheumatoid arthritis (RA) [Abstract]. Arth Rheum 44[supp 9]S106

[r24] Marshall JD Brauer M Frank LD 2009 Healthy neighborhoods: walkability and air pollution. Environ Health Perspect 117 1752 1759; 10.1289/ehp.0900595 20049128 PMC2801167

[r25] Marshall JD, Nethery E, Brauer M (2008). Within-urban variability in ambient air pollution: comparison of estimation methods.. Atmos Environ.

[r26] National Center for Health Statistics. (1998). International Classification of Diseases, Ninth Revision (ICD-9).. ftp://ftp.cdc.gov/pub/Health_Statistics/NCHS/Publications/ICD-9/ucod.txt.

[r27] Omara FO, Fournier M, Vincent R, Blakley BR (2000). Suppression of rat and mouse lymphocyte function by urban air particulates (Ottawa dust) is reversed by *N*-acetylcysteine.. J Toxicol Environ Health A.

[r28] Orsini N, Bellocco R, Bottai M, Wolk A, Greenland S (2008). A tool for deterministic and probabilistic sensitivity analysis of epidemiologic studies.. Stata J.

[r29] Parks CG, Conrad K, Cooper GS (1999). Occupational exposure to crystalline silica and autoimmune disease.. Environ Health Perspect.

[r30] Prasher D (2009). Is there evidence that environmental noise is immunotoxic?. Noise Health.

[r31] Rogers MP, Trentham DE, Dynesius-Trentham R, Daffner K, Reich P (1983). Exacerbation of collagen arthritis by noise stress.. J Rheumatol.

[r32] Sarnat JA, Schwartz J, Catalano PJ, Suh HH (2001). Gaseous pollutants in particulate matter epidemiology: confounders or surrogates?. Environ Health Perspect.

[r33] Setton EM, Hystad PW, Keller CP. (2005). Road Classification Schemes—Good Indicators of Traffic Volume? UVIC SSL Working Paper 05–014. Spatial Sciences Laboratories Occasional Papers Series 2005.. http://web.uvic.ca/~ssrl01/SSRLtemp/SSL05-014-TRAFFIC.pdf.

[r34] Singh JA, Holmgren AR, Noorbaloochi S (2004). Accuracy of Veterans Administration databases for a diagnosis of rheumatoid arthritis.. Arthritis Rheum.

[r35] Statistics Canada. (2001). Census of Canada. Government of Canada.. http://www12.statcan.ca/english/census01/home/index.cfm.

[r36] Statistics Canada. (2002). Canadian Community Health Survey (CCHS) Detailed information for 2000–2001 (Cycle 1.1).. http://www23.statcan.gc.ca/imdb/p2SV.pl?Function=getSurvey&SurvId=1630&InstaId=3359&SDDS=3226.

[r37] Sugiyama D, Nishimura K, Tamaki K, Tsuji G, Nakazawa T, Morinobu A (2010). Impact of smoking as a risk factor for developing rheumatoid arthritis: a meta-analysis of observational studies.. Ann Rheum Dis.

[r38] Transport Canada. (2005). TP1247–Aviation–Land Use in the Vicinity of Airports.. http://www.tc.gc.ca/eng/civilaviation/publications/tp1247-menu-1418.htm.

[r39] University of British Columbia. (2014). Border Air Quality Study.. http://baqs.spph.ubc.ca/border-air-quality-study/welcome-border-air-quality-study.

[r40] Walitt BT, Constantinescu F, Katz JD, Weinstein A, Wang H, Hernandez RK (2008). Validation of self-report of rheumatoid arthritis and systemic lupus erythematosus: the Women’s Health Initiative.. J Rheumatol.

[r41] Wang R, Henderson SB, Sbihi H, Allen RW, Brauer M (2012). Temporal stability of land use regression models for traffic-related air pollution.. Atmos Environ.

[r42] Zaharna M, Guilleminault C (2010). Sleep, noise and health: review. Noise Health.

