# Supplementary material for: Proximity to Traffic, Ambient Air Pollution, and Community Noise in Relation to Incident Rheumatoid Arthritis
Source: Environ Health Perspect. 2014 Jun 6;122(10):1075–80. doi: 10.1289/ehp.1307413 (PMC4181921; doi:10.1289/ehp.1307413)
Supplement: (264 KB) PDF [file ehp.1307413.s001.508.pdf]

Environmental Health Perspectives doi:10.1289/ehp.1307413

Supplemental Material

Proximity to Traffic, Ambient Air Pollution, and Community Noise in Relation to Incident Rheumatoid Arthritis

Anneclaire J. De Roos, Mieke Koehoorn, Lillian Tamburic, Hugh W. Davies, and Michael Brauer

Table of Contents **Page**

Table S1. Air pollution and noise exposure distributions by residential proximity to traffic in the at-risk cohort during the cohort definition period (1994-1998) 2

Table S2. Risk of incident RA in relation to NO and NO_2, assessed using inverse distance weighting (IDW) (odds ratios [OR] and 95% confidence intervals [CI] for interquartile range [IQR] increase) 3

Table S3. Risk of incident RA in relation to ambient air pollution and community noise (odds ratios [OR] and 95% confidence intervals [CI] for quintiles of exposure) 4

Table S1. Air pollution and noise exposure distributions by residential proximity to traffic in the at-risk cohort during the cohort definition period (1994-1998).[a]

Exposure	NO-LUR (μg/m^3)	NO_2-LUR (μg/m^3)	Black carbon-LUR (μg/m^3)	$PM_{2.5}$-LUR (μg/m^3)	PM_{10}-IDW (μg/m^3)	O_3-IDW (μg/m^3)	CO-IDW (μg/m^3)	SO_2-IDW (μg/m^3)	Noise (dB(A))
No. observations	640,041	640,041	616,672	620,535	499,015	586,777	501,888	590,348	445,887
**Highway**									
Never lived near highway	29.75 ± 10.50	28.69 ± 4.97	1.24 ± 0.60	4.65 ± 2.32	13.90 ± 0.57	27.10 ± 4.91	739.4 ± 152.7	6.39 ± 2.73	62.99 ± 4.77
>50m to 150m from highway	36.43 ± 14.30	31.69 ± 5.77	1.63 ± 0.64	5.46 ± 2.51	13.78 ± 0.51	26.30 ± 5.39	765.2 ± 160.7	7.26 ± 3.03	67.36 ± 5.07
≤50m from highway	44.95 ± 17.74	33.51 ± 5.91	1.60 ± 0.61	5.41 ± 2.78	13.71 ± 0.50	28.26 ± 5.62	725.7 ± 171.8	6.31 ± 3.06	71.33 ± 5.17
**Major road**									
Never lived near major road	26.73 ± 7.12	27.37 ± 4.38	1.17 ± 0.56	4.39 ± 2.22	13.91 ± 0.56	27.72 ± 4.78	719.9 ± 146.7	6.10 ± 2.60	62.29 ± 4.71
>50m to 150m from major road	33.64 ± 11.71	31.78 ± 5.30	1.41 ± 0.65	5.40 ± 2.46	13.82 ± 0.56	25.64 ± 5.04	782.5 ± 158.5	7.19 ± 2.94	64.00 ± 4.59
≤50m from major road	44.18 ± 15.91	31.60 ± 5.15	1.53 ± 0.65	4.98 ± 2.45	13.91 ± 0.60	27.14 ± 5.08	739.9 ± 155.5	6.51 ± 2.85	67.98 ± 5.20

Abbreviations: LUR=land-use regression; IDW=inverse-distance weighting

[a]Members of the study population who lived within 150m from a highway/major road during the 5-year exposure period were categorized according to proximity at which they lived the longest.

Table S2. Risk of incident RA in relation to NO and NO_2, assessed using inverse distance weighting (IDW) (odds ratios [OR] and 95% confidence intervals [CI] for interquartile range [IQR] increase).[a]

Exposure	RA-ICD9: Case N	RA-ICD9: Control N	RA-ICD9: OR (95% CI) [IQR]	RA-prescription: Case N	RA-prescription: Control N	RA-prescription: OR (95% CI) [IQR]	RA-specialist: Case N	RA-specialist: Control N	RA-specialist: OR (95% CI) [IQR]
NO-IDW (μg/m^3)	2554	25677	0.82 (0.78, 0.87) [13.9]	1992	20659	0.81 (0.76, 0.87) [13.7]	1535	14816	0.81 (0.75, 0.87) [13.9]
NO_2-IDW (μg/m^3)	2560	25739	0.83 (0.78, 0.87) [9.1]	1994	20692	0.81 (0.76, 0.86) [8.9]	1536	14840	0.82 (0.76, 0.88) [9.1]

Abbreviations: RA-ICD9=case definition based on 3 ICD9 codes; RA-prescription=case definition based on 2 ICD9 codes plus prescription; RA-specialist=case definition based on 2 ICD9 codes plus visit to specialist; IQR=interquartile range; IDW=inverse-distance weighting

[a]Odds ratios are adjusted for age, sex, and neighborhood socioeconomic status

Table S3. Risk of incident RA in relation to ambient air pollution and community noise (odds ratios [OR] and 95% confidence intervals [CI] for quintiles of exposure).[a]

Exposure	RA-ICD9: Case N	RA-ICD9: Control N	RA-ICD9: OR (95% CI)	RA-prescription: Case N	RA-prescription: Control N	RA-prescription: OR (95% CI)	RA-specialist: Case N	RA-specialist: Control N	RA-specialist: OR (95% CI)
**NO-LUR**	3280	33234		2659	26846		1883	19059	
1st quintile			*Referent*			*Referent*			*Referent*
2nd quintile			0.97 (0.86, 1.08)			1.05 (0.93, 1.19)			0.95 (0.82, 1.10)
3rd quintile			0.94 (0.84, 1.06)			0.98 (0.86, 1.11)			0.94 (0.81, 1.09)
4th quintile			0.92 (0.82, 1.03)			0.90 (0.79, 1.03)			0.82 (0.71, 0.96)
5th quintile			0.96 (0.85, 1.08)			0.92 (0.80, 1.05)			0.86 (0.74, 1.00)
**NO-IDW**	2554	25677		1992	20659		1535	14816	
1st quintile			*Referent*			*Referent*			*Referent*
2nd quintile			0.87 (0.77, 0.99)			0.88 (0.76, 1.01)			0.90 (0.76, 1.06)
3rd quintile			0.86 (0.76, 0.98)			0.87 (0.76, 1.01)			0.99 (0.84, 1.16)
4th quintile			0.78 (0.69, 0.89)			0.83 (0.72, 0.96)			0.80 (0.67, 0.94)
5th quintile			0.67 (0.58, 0.76)			0.64 (0.55, 0.75)			0.65 (0.54, 0.77)
**NO_2-LUR**	3278	33229		2657	26842		1881	19059	
1st quintile			*Referent*			*Referent*			*Referent*
2nd quintile			0.92 (0.82, 1.03)			0.93 (0.82, 1.05)			0.85 (0.73, 0.98)
3rd quintile			0.84 (0.75, 0.95)			0.94 (0.83, 1.06)			0.87 (0.75, 1.00)
4th quintile			0.90 (0.80, 1.01)			0.82 (0.72, 0.93)			0.82 (0.71, 0.95)
5th quintile			0.97 (0.86, 1.08)			0.76 (0.67, 0.87)			0.77 (0.66, 0.90)
**NO_2-IDW**	2560	25739		1994	20692		1536	14840	
1st quintile			*Referent*			*Referent*			*Referent*
2nd quintile			0.87 (0.77, 0.98)			0.96 (0.83, 1.10)			1.03 (0.87, 1.20)
3rd quintile			0.80 (0.70, 0.90)			0.90 (0.78, 1.04)			0.97 (0.82, 1.14)
4th quintile			0.76 (0.67, 0.86)			0.74 (0.63, 0.85)			0.76 (0.64, 0.90)
5th quintile			0.64 (0.56, 0.73)			0.60 (0.51, 0.70)			0.63 (0.53, 0.76)

Exposure	RA-ICD9: Case N	RA-ICD9: Control N	RA-ICD9: OR (95% CI)	RA-prescription: Case N	RA-prescription: Control N	RA-prescription: OR (95% CI)	RA-specialist: Case N	RA-specialist: Control N	RA-specialist: OR (95% CI)
**Black carbon-LUR**	3138	32159		2553	25935		1818	18420	
1st quintile			*Referent*			*Referent*			*Referent*
2nd quintile			1.13 (1.01, 1.27)			1.13 (0.99, 1.28)			1.08 (0.93, 1.25)
3rd quintile			1.00 (0.88, 1.12)			1.02 (0.90, 1.16)			0.99 (0.85, 1.16)
4th quintile			1.02 (0.91, 1.15)			0.93 (0.81, 1.06)			0.91 (0.78, 1.06)
5th quintile			0.94 (0.83, 1.06)			0.91 (0.80, 1.05)			0.86 (0.73, 1.01)
**$PM_{2.5}$-LUR**	3175	32304		2567	26144		1819	18518	
1st quintile			*Referent*			*Referent*			*Referent*
2nd quintile			0.98 (0.87, 1.10)			0.95 (0.83, 1.07)			0.90 (0.78, 1.05)
3rd quintile			0.90 (0.80, 1.01)			0.88 (0.77, 1.00)			0.86 (0.74, 1.00)
4th quintile			0.93 (0.83, 1.05)			0.91 (0.80, 1.04)			0.86 (0.74, 1.00)
5th quintile			0.91 (0.80, 1.02)			0.83 (0.72, 0.95)			0.80 (0.68, 0.94)
**PM_{10}-IDW**	2712	27208		2135	21850		1653	15709	
1st quintile			*Referent*			*Referent*			*Referent*
2nd quintile			0.84 (0.74, 0.94)			0.87 (0.75, 0.99)			0.92 (0.79, 1.08)
3rd quintile			0.80 (0.71, 0.91)			0.78 (0.67, 0.90)			0.83 (0.71, 0.98)
4th quintile			0.75 (0.66, 0.85)			0.73 (0.63, 0.84)			0.77 (0.65, 0.91)
5th quintile			0.66 (0.57, 0.76)			0.69 (0.59, 0.81)			0.74 (0.62, 0.89)
**O_3-IDW**	3055	30698		2454	24791		1724	17636	
1st quintile			*Referent*			*Referent*			*Referent*
2nd quintile			1.15 (1.02, 1.30)			1.33 (1.16, 1.53)			1.37 (1.16, 1.62)
3rd quintile			1.10 (0.97, 1.24)			1.31 (1.14, 1.51)			1.39 (1.18, 1.64)
4th quintile			1.29 (1.14, 1.45)			1.54 (1.34, 1.77)			1.55 (1.32, 1.83)
5th quintile			1.29 (1.14, 1.45)			1.56 (1.35, 1.79)			1.08 (0.91, 1.28)
**CO-IDW**	2826	28269		2249	22807		1633	16274	
1st quintile			*Referent*			*Referent*			*Referent*
2nd quintile			0.95 (0.84, 1.07)			1.00 (0.88, 1.14)			0.93 (0.79, 1.09)
3rd quintile			0.81 (0.71, 0.91)			0.86 (0.75, 0.99)			0.90 (0.77, 1.05)
4th quintile			0.82 (0.73, 0.93)			0.80 (0.69, 0.92)			0.86 (0.73, 1.02)
5th quintile			0.75 (0.66, 0.85)			0.68 (0.59, 0.78)			0.70 (0.59, 0.82)

Exposure	RA-ICD9: Case N	RA-ICD9: Control N	RA-ICD9: OR (95% CI)	RA-prescription: Case N	RA-prescription: Control N	RA-prescription: OR (95% CI)	RA-specialist: Case N	RA-specialist: Control N	RA-specialist: OR (95% CI)
**SO_2-IDW**	3082	30963		2477	25011		1733	17761	
1st quintile			*Referent*			*Referent*			*Referent*
2nd quintile			1.11 (0.98, 1.25)			1.16 (1.02, 1.33)			0.98 (0.83, 1.15)
3rd quintile			1.11 (0.98, 1.24)			1.10 (0.97, 1.26)			1.24 (1.06, 1.44)
4th quintile			0.93 (0.83, 1.05)			0.91 (0.79, 1.04)			0.93 (0.79, 1.09)
5th quintile			0.85 (0.75, 0.96)			0.74 (0.64, 0.85)			0.82 (0.70, 0.97)
**Noise**	2188	22734		1711	18346		1315	13173	
1st quintile			*Referent*			*Referent*			*Referent*
2nd quintile			0.99 (0.86, 1.14)			0.85 (0.72, 1.00)			0.91 (0.76, 1.09)
3rd quintile			0.91 (0.79, 1.05)			0.94 (0.80, 1.10)			0.78 (0.65, 0.93)
4th quintile			1.02 (0.88, 1.17)			0.94 (0.81, 1.11)			0.95 (0.79, 1.13)
5th quintile			1.06 (0.92, 1.22)			0.94 (0.80, 1.10)			0.88 (0.74, 1.06)

Abbreviations: RA-ICD9=case definition based on 3 ICD9 codes; RA-prescription=case definition based on 2 ICD9 codes plus prescription; RA-specialist=case definition based on 2 ICD9 codes plus visit to specialist; LUR=land-use regression; IDW=inverse-distance weighting

[a]Odds ratios are adjusted for age, sex, and neighborhood socioeconomic status.
